# Outdoor air pollution from industrial chemicals causing new onset of asthma or COPD: a systematic review protocol

**DOI:** 10.1186/s12995-020-00289-6

**Published:** 2020-12-28

**Authors:** Harald Lux, Xaver Baur, Lygia Therese Budnik, Astrid Heutelbeck, João Paulo Teixeira, Emeri Neumann, Diana Adliene, Judita Puišo, David Lucas, Jakob Löndahl, Athanasios Damialis, Ozlem Goksel, Hans Orru

**Affiliations:** 1 Occupational, Social and Environmental Medicine, University Hospital Jena – Friedrich Schiller University Jena, Erlanger Allee 103, 07747 Jena, Germany; 2 Department of Psychiatry and Psychotherapy, Ruppiner Kliniken, Neuruppin, Germany; 3 European Society for Environmental and Occupational Medicine, Berlin, Germany; 4 Emeritus University of Hamburg, Hamburg, Germany; 5 Translational Toxicology and Immunology Unit, Institute for Occupational and Maritime Medicine, University Medical Center Hamburg-Eppendorf, Hamburg, Germany; 6 Environmental Health Department, National Institute of Health, Porto, Portugal; 7 EPIUnit - Instituto de Saúde Pública, Universidade do Porto, Porto, Portugal; 8 Institute of Family Medicine and Public Health, University of Tartu, Tartu, Estonia; 9 Department of Physics, Kaunas University of Technology, Kaunas, Lithuania; 10 EA4324 ORPHY Laboratory, Occidental Brittany University Brest, Brest, France; 11 Ergonomics and Aerosol Technology, Lund University, Lund, Sweden; 12 Chair and Institute of Environmental Medicine, UNIKA-T, Technical University of Munich and Helmholtz Centre Munich, Augsburg, Germany; 13 Laboratory of Occupational & Environmental Respiratory Diseases, Division of Immunology, Allergy and Asthma, Department of Pulmonary Medicine, Faculty of Medicine, EGE University, Izmir, Turkey; 14 Section of Sustainable Health, Umea University, Umea, Sweden

**Keywords:** Chronic obstructive pulmonary disease, Outdoor exposure, Occupational exposure, Air pollution, Systematic literature review, Respiratory disease, Inhaled particles

## Abstract

**Background:**

Until today, industrial sources contribute to the multifaceted contamination of environmental air. Exposure to air pollutants has the potential to initiate and promote asthma and chronic obstructive pulmonary disease (COPD). At global scale, both entities cause the majority of about 4 million annual deaths by respiratory disease. However, we identified industrial contamination as a subgroup of air pollution that may be associated with this burden and is underinvestigated in research. Therefore, the aim of this study is to investigate associations between substances industrially released into environmental air and the occurrence of asthma and COPD in the human population. Here we present the protocol for our systematic review of the current evidence.

**Methods:**

The following determinations will be applied during the systematic review process and are specified in the protocol that complies with the PRISMA-P statement. Populations of children and adults, as well as outdoor workers, exposed to industrially released air pollutants are of interest. Eligible studies may include subjects as controls who are non- or less exposed to the investigated air pollutants. The outcomes new-onset asthma and/or COPD investigated with risk ratio, odds ratio, hazard ratio, incidence rate ratio, cumulative incidence, and incidence rate are eligible. We will search the electronic literature databases EMBASE, MEDLINE, and Web of Science for peer-reviewed reports of incidence studies and incidence case-control studies. After systematic sorting of initial records, included studies will be subjected to quality assessment. Data will be synthesized qualitatively and, if appropriate, quantitatively for risk ratio and odds ratio. We will maintain and provide a PRISMA report.

**Discussion:**

Results of this systematic review may indicate alterations of incidence and risk of asthma and/or COPD in populations within industrial exposure radiuses including outdoor workplaces. Specific causal substances and compositions will be identified, but results will depend on the exposure assessment of the eligible studies. Our approach covers effects of industrial contributions to overall air pollution if studies reportedly attribute investigated emissions to industry. Results of this study may raise the question wether the available higher-level evidence sufficiently covers the current scale of industrial exposure scenarios and their potential harm to respiratory health.

**Trial registration:**

This protocol was registered in PROSPERO, registration number CRD42020151573.

This protocol bases on the guideline of PRISMA-P [1].

## Administrative information

### Registration

This protocol was registered in PROSPERO under the registration number CRD42020151573.

### Amendments

Amendments of the protocol will be described and documented in PROSPERO and in the PRISMA-report [2]. We will provide the report to the reader as supplementary material of the final systematic review article.

### Support

#### Sources

This systematic review is funded by EU-COST Action DiMoPEx (funding reference number CA-15129; to LTB). The COST action is supported by the EU Framework Programme Horizon 2020. HO’s work is supported by the Estonian Ministry of Education and Research Grant IUT34–17. JL’s contribution is funded within a project by the Swedish Research Council for Health, Working Life and Welfare (FORTE, number 2017–00690).

#### Role of funder

We planned two short-term scientific missions financed by EU-COST. The two authors receiving the grants will carry out tasks in the process of this systematic review.

The chair of EU-COST Action DiMoPEx LTB participated in the preparation of the protocol.

## Introduction

### Rationale

According to the World Health Organization 41 million people died from noncommunicable diseases (NCD) worldwide in 2016 which represents 71% of all global deaths [3]. The respiratory tract belongs to the mostly involved organs. In particular, asthma and COPD had the major impact on the overall worldwide mortality of 4.2 million caused by respiratory diseases in 2008 [4]. Besides mortality, the chronic nature of asthma and COPD causes considerable morbidity. 6.62 million disability-adjusted life years (DALY) for COPD alone worldwide in 2012 [5] indicate an immense socio-economic impact of noncommunicable respiratory diseases.

Environmental air pollution contributes to this huge burden through the initiation and promotion of respiratory diseases that are leading causes of death [4].

An important cause of these NCDs is industrial air pollution. However, its quantity and compositions vary considerably by localization. As an example, living in the proximity to industrial facilities seems to be an adverse condition. Higher morbidity for new-onset asthma in the general population is reported for children who live near petrochemical production facilities [6]. Furthermore, the impact of outdoor NO_2_, PM_2.5_ and black carbon on childhood asthma incidence is estimated to be significant [7]. A meta-analysis demonstrated statistically significant associations for traffic-related air pollution and risk of asthma development in children [8]. Besides traffic, industrial processes including oil and energy production can release considerable amounts of contributing hazardous and different pollutants.

COPD alone was considered responsible for 242.250 deaths worldwide due to ambient air pollution in 2012 [5]. This highlighted the necessity for the detection of different origins of airborne environmental exposure and its particular respiratory health effects.

Furthermore, the occupational population is confronted with specific risks for both asthma and COPD [9, 10]. Diisocyanates, formaldehyde, anhydrides and metal salts are examples for airborne chemicals that induce asthma and sometimes COPD [11]. Moreover, inorganic or organic dust or fumes, e.g., mine and cement dust, trigger COPD [11].

Residents and workers in the micro-environments of outdoor occupations, such as harbors or petrochemical sites, can be close to these and other potentially causal agents. Certain exposures are similarly present in industrial workplaces and their neighborhood, sometimes also in the general environment. Evidence from outdoor occupational settings provides important information about possible respiratory health risks for the general population.

So far, a comprehensive systematic review of the current contribution of airborne industrial agents to the morbidity of asthma and COPD has been lacking; the adverse effects of these agents on respiratory health require further investigation.

The hypothesis is that exposure to environmental industrial air pollution causes new-onset asthma and/or COPD in the general population of adults and children. There is also evidence that several environmentally released substances cause new-onset asthma and/or COPD in outdoor occupational settings (e.g., near harbors or petrochemical sites), where these substances can occur in high concentrations.

Depending on the extent of studies that will be detected according to this protocol, the publication may be split in two separate articles for COPD and asthma.

### Objectives

This systematic review aims at evaluating the evidence of the effect of outdoor industrial air pollution on the occurrence of new-onset asthma and COPD. We refer to PECO, the PICO [12] approach adapted to studies on exposure effects for the structure of our review questions.

1. Are adults and/or children of the general population who are exposed to industrial environmental air pollution at a higher risk of new-onset asthma than adults and/or children in control groups comprising subjects who are not exposed or less exposed to this pollution?
2. Which are the industrially released airborne substances in environmental air that may cause asthma?
3. Are adults with outdoor occupational exposure to the substances that are investigated in the general population at a higher risk of new-onset asthma than adults in control groups comprising subjects who are not exposed or less exposed to this pollution?
4. Are adults and/or children of the general population who are exposed to industrial environmental air pollution at a higher risk of new-onset COPD than adults and/or children in control groups comprising subjects who are not exposed or less exposed to this pollution?
5. Which are the industrially released airborne substances in environmental air that may cause COPD?
6. Are adults with outdoor occupational exposure to the substances that are investigated in the general population at a higher risk of new-onset COPD than adults in control groups comprising subjects who are not exposed or less exposed to this pollution?

#### Population

The population includes adults and children in the environmental field and workers in outdoor occupational settings.

#### Exposure

The exposure is industrially caused environmental airborne chemicals. Exposure in outdoor occupational settings is of interest, when an effect on the population in the general environment was investigated.

#### Comparison

The accepted comparator will be humans who are not exposed or who are exposed to lower levels of airborne substances than exposed subjects are. With the broad range of potentially causal substances and exposure assessment methods no further specifications are pre-defined.

#### Outcome

The outcomes are new-onset asthma and COPD measured with the defined diagnostic methods. We will consider incidence and measures of effect based on incidence of the diseases of interest.

Details are provided in the following section about eligibility criteria.

## Methods

### Eligibility criteria

Studies meeting the following criteria will be selected.

#### Study design

Studies investigating effects of industrial environmental air pollution or exposure in outdoor occupational settings on new-onset asthma and/or COPD often use cross-sectional study designs based on prevalence [6, 13]. The practicability of cross-sectional analyses for investigations of newly suspected causative factors and the lower demand of resources compared to incidence-based analyses might cause this trend [14]. Evidence from prevalence-based studies might be sensitive, because results for emerging and less studied threats in an earlier stage of risk-evaluation would be included. However, we define studies based on prevalence, respectively cross-sectional studies, lacking an observation period for the registration of new-onset disease, as ineligible. Consequently, case series, and case reports are also excluded.

The most appropriate observational study designs to indicate a causal relationship between exposure and effect are incidence-based [14]. Therefore, we will include incidence studies (e.g., cohort studies) and incidence case-control studies [14]. Inclusion of this higher level of evidence may lead to the most specific and sound results.

Systematic reviews and meta-analyses are ineligible. However, if relevant for the objective, we will keep them for the screening of reference lists.

#### Time frame

Articles of the time period from 2000 to the current year are of interest. The reason for this limitation is changes in regulations and production processes over time. We expect them to have an impact on quality and quantity of substances released into environmental air. We determined studies that have been published within the last 20 years as being representative for the exposures that might be currently relevant and comparable.

#### Population

The eligible population is adults in the environmental and in the outdoor occupational field. Children are eligible in the environmental field. Workers are included when the outdoor workplace is attributable to industry and according to the exposure of interest.

Populations are eligible when at least a part is reportedly exposed to the pollutants of interest. All subjects or groups need to have a known exposure status.

#### Exposure

Airborne exposure to chemicals released from industrial processes is eligible. Examples are petrochemicals, polycyclic aromatic hydrocarbons (PAH), formaldehyde, chlorine, ammonia, nitric oxides, isocyanates, acid anhydrides and metals (metal salts). Man-made mineral matter (e.g., cement dust) is also included. Chemical pollution caused by fertilizers or processed organic matter, such as ammonia from manure is included. However, allergens, e.g., pollen, fungi, and the common allergens in workplaces (e.g., wheat flour, latex, animal dander and enzymes) are not eligible. Naturally occurring minerals, such as silica and asbestos, are also excluded. Radiation and exposure to chemical warfare agents in bellicose conflicts are ineligible. Particular matter (PM) of unspecified constituents and gaseous emissions are explicitly included. The definition of the term “industry” used in this systematic review is: “companies and activities involved in the process of producing goods for sale, especially in a factory or special area” [15]. In this sense, the authors explicitly include the branches agriculture, construction, and electricity generation.

Environmental exposure is eligible when it is caused by the release of airborne pollutants from industrial processes into environmental air.

There are no limitations to the method of exposure assessment for eligibility. However, data based on actual measurements are desirable and of higher quality, i.e., personal air monitoring or ambient air monitoring. Studies can also determine exposure statuses based on geographic proximity to a known source of industrial air pollution. This would be a surrogate for measurement data, which relies on the assumption that quantities of pollutants are higher close to industrial sources and lower further away. Confirmation of nearby sources of pollution by questionnaire data is possible. Environmental modeling that can use different kinds of data with various methods is accepted. Occupational exposure is eligible when airborne substances in outdoor workplaces are also found in environmental air. Consequently, available studies on health effects in the environment determine the eligibility of studies on substances in the occupational field (e.g., formaldehyde).

Presence and/or concentration of exposure in the air of workplaces have to be monitored. Alternatively, the presence of substances in a workplace must be apparent, because exposure to a certain material of interest was verified under comparable conditions, e.g., asphalt paving in road construction. Biomonitoring is not obligatory.

If the exposure is mixed, e.g., dusts or complete emission of industrial origin, but contains eligible substances, the study will be included.

Household exposure is not defined as industrial in this review and therefore excluded. Examples are fuel aerosols released by domestic cooking as well as cooking in the streets, use of domestic chemicals and household products.

Traffic air pollution in public spaces is not included in the scope of this review, despite a share that might be attributable to industrial activities (e.g., transport of materials and products).

If a study reports effects of chemicals that usually enter the human organism in a different way than via the respiratory tract and respiratory exposure is not proven and mentioned (e.g., Bisphenol A), the study will be excluded.

We refer to exposure that is present in outdoor air and that is not enclosed resp. confined to compartments, e.g., in buildings, with the term “environmental exposure”. The term “environmental air” corresponds to outdoor air in the same sense throughout this protocol.

#### Comparison

For the estimation of measures of effect, cohort studies may compare a group of subjects exposed to industrial air pollution with a control-group of non-exposed subjects. Sometimes, a control-group consists of subjects exposed to lower levels of the investigated pollution than the exposed group. Case-control studies also require exposure statuses assigned to all subjects for the estimation of effect measures. Usually, the statuses are exposure and no exposure among both cases and controls. However, comparisons are not required for eligibility, i.e. if a study reports incidence. Moreover, with the broad range of potentially causal substances and methods of exposure assessment, controls in studies of interest may have different characteristics. Therefore, specific characteristics of the comparisons, e.g., certain cut-off values, are inconsiderable for eligibility. However, selection and comparability of controls are part of the quality assessment.

#### Statistical measures

Risk ratio, odds ratio, hazard ratio, incidence rate ratio, cumulative incidence, and incidence rate of new-onset asthma and COPD are the eligible statistical measures.

#### Diagnosis of the condition of interest

We determine the following criteria and references for the diagnosis of the conditions of interest new-onset asthma and COPD.

We determine studies that rely on diagnosis of asthma according to the ERS, ATS and/or GINA guidelines [16–18] to be of high quality concerning diagnosis.

Diagnosis of COPD according to the leading international guidelines of the ERS, ATS and GOLD [19, 20] for adults and children is likewise determined as high quality.

For example, a forced expiratory volume in 1 s over forced vital capacity ratio (FEV1/FVC) below the 5th percentile, the lower limit of normal (LLN) and the equivalent to a z-score of − 1.645, is the diagnostic criterion of ERS/ATS distinguishing between a healthy and potentially pathologic lung function [16]. A post-bronchodilator FEV1/FVC below 70% of the predicted ratio is the limit for a diagnosis of COPD according to GOLD [16, 19].

There may be particular studies that do not explicitly mention these references. In such cases, we accept reporting of the diagnostic measures and limits that the aforementioned guidelines demand as equivalent.

The reference of a particular study to a diagnostic standard will be traced to a previous publication if indicated.

Diagnosis of asthma and COPD by a physician is the minimum diagnostic criterion for inclusion in qualitative and/or quantitative analysis. Studies using data in accordance with the aforementioned criteria provided by registries or collected with questionnaires are explicitly eligible. Registries may use appropriate case definitions for asthma and COPD, and questionnaires may ask for diagnoses by a physician. Definitions of the outcomes that are based on symptoms alone are not accepted for inclusion.

#### Report characteristics

The language of included reports is English. Articles in other languages than English will not be processed.

Articles need to be published in indexed (MEDLINE, EMBASE or Web of Science) scientific journals and subjected to peer review.

We exclude grey literature (conference abstracts/proceedings, theses, etc.), letters to the editor, and unpublished data. If comments or letters to the editor refer to eligible studies, they will be included as adjuncts to the particular article.

### Information sources

We will develop electronic literature search strategies using MeSH terms (Medical subject headings) and text words. The electronic searches will be applied to MEDLINE (OVID interface, 2000 onwards), EMBASE (OVID interface, 2000 onwards), and Web of Science (2000 onwards). The literature search will be limited to human subjects and articles in the English language.

We will scan the reference lists of the articles undergoing full-text screening for inclusion/exclusion for relevant articles in order to ensure coverage of the relevant literature. Likewise, we will scan the reference lists of relevant systematic reviews and meta-analyses detected by the electronic search.

Articles from collections of the authors that are identified as potentially relevant can also be introduced into the study selection process.

### Search strategy

We will search quantitative studies that include the eligible statistical parameters. No restriction of study types is included into the electronic search strategy. Validated filters, preferably functions provided by the databases, for studies on human subjects and English full texts will be applied.

The search period from 2000 to the current year will be implemented and the search be updated after 12 months.

We provide the full electronic search strategy in the supplementary material.

Additional to manual sorting, a validated filter of the SIGN initiative will help to identify systematic reviews and meta-analyses among initial search results [21].

### Study records

#### Data management

References of literature search results will be imported into a citation software, cleared from duplicates, and saved in an export format for documentation. We will use the software Rayyan [22] or an alternative for screening purposes and manage the systematic review tasks with appropriate document formats and folders. We will retrieve and store the required full texts. Full texts and the required forms for inclusion/exclusion, data extraction, and bias and quality assessment will be distributed to the reviewers for collaborative work.

#### Selection process

We will identify and remove duplicate references from the initial search results of the three databases. For this purpose, we will use functions of the reference management software EndNote and the screening management software Rayyan [22].

We will screen all titles and abstracts of the extracted references for relevance independently and in duplicate.

One reviewer will screen all records alone and a group of the remaining reviewers will split the records for a parallel screening round. Initial selections of the latter will be further evaluated by other reviewers involved in the parallel screening. Conflicts will be discussed until an agreement is reached.

All records that will be selected by both parallel screenings will undergo the subsequent process for study inclusion.

The references of studies on occupational exposure will be checked for a match with studies on environmental exposure. Studies investigating occupational exposure of the same type as studies on environmental exposure will be kept. However, studies on exposures to substances exclusively investigated in the occupational field will be excluded.

The full-text inclusion assessment of all remaining articles from title/abstract screening will be performed using a self-developed form. We will pilot the inclusion form and apply the final version to the full texts. Two reviewers will assess independently and in duplicate allocated packages of the full texts for eligibility. If discrepancies persist after being discussed by the two reviewers, a third reviewer will decide. This reviewer must have the best expertise on the subject among the remaining reviewers. If necessary, the whole group will discuss the discrepancy. After the full-text inclusion assessment, the remaining reports will undergo a duplicate reporting check.

#### Data collection process

We will develop an extraction form and pilot it. One reviewer will independently extract data from the included articles using the final form. A second reviewer, who works with the allocated package of included studies, will retrace extracted data and discuss discrepancies until consensus is reached. If the two reviewers do not reach consensus, a third reviewer will decide. This reviewer must have the best expertise on the subject among the remaining reviewers. If necessary, the whole group will discuss the discrepancy.

### Data items

We provide this list of information that we plan to seek.

Identification of the study:

- title
- ID number
- year of publication
- journal
- authors

Characteristics of the study population:

- number of included individuals
- age
- sex
- weight
- body height
- health status, i.e. co-morbidity
- stage, resp. severity of the disease
- disease entity and/or syndrome
- social or cultural characteristics (e.g., education level and income)
- occupation
- ethnicity
- geographic information
- symptoms
- risk factors for COPD or asthma (including smoking)
- level of physical activity

Outcomes:

- Definition of the diagnosis of asthma and COPD, including references to guidelines (ATS/ERS, GINA, GOLD) and the specific diagnostic criteria (in particular with FEV1/FVC below the LLN or post-bronchodilator FEV1/FVC < 70% of the predicted ratio for COPD).

Study details, methods and results:

- type of study
- start and end of the study
- study duration
- inclusion and exclusion criteria for participation
- number of exposed groups and comparison groups
- number of participants in each group
- absolute and relative frequencies
- quantification and explanation of withdrawals and exclusions
- duration of follow-up
- statistical method that was used
- statistical measures risk ratio, odds ratio, hazard ratio, incidence rate ratio, cumulative incidence, and incidence rate
- standard error
- sample size
- confidence interval
- number of confounders the calculation of the estimation was adjusted for
- quality of the particular confounders if provided
- baseline risk

Exposure:

- description of the substance(s) and or mixtures
- method of exposure assessment
- the concentration(s) of substances or mixtures the populations are exposed to or the distance, resp. spatial relationship of the study population to the source of industrial pollution
- description of the industrial source of the substance(s) or mixtures
- duration of exposure
- co-exposures
- use of personal breathing protection

Other:

- country the study was conducted in
- conflict of interest
- funding source
- key conclusions of the study authors
- issues affecting directness

### Outcomes and prioritization

New-onset of asthma and COPD are our primary outcomes to be collected. This choice is based on the methodologic adequacy of estimations using incidence of disease for investigating associations between exposure and disease.

### Risk of bias in individual studies

We will apply the Newcastle Ottawa Scale (NOS) [23] for the assessment of risk of bias on the individual study level. A table containing all ratings will be provided. If we become aware of a more suitable method, we may switch to the alternative. Resulting information on the risk of bias due to selection, exposure and outcome, e.g., the diagnostic standard, will be used for the exploration of heterogeneity in the data synthesis.

### Data synthesis

#### Quantitative synthesis

We will evaluate the adequacy of included studies for quantitative analysis. Exposure, study design and comparison need to be sufficiently homogeneous for the conduction of a meta-analysis. Risk ratio and odds ratio may be quantitatively synthesized. Besides these effect measures, reports must must provide data for the calculation of the standard error (sample size, absolute and relative frequencies, confidence interval). We will apply RR and OR simultaneously. For prevalence less than 10%, OR and RR are approximately equal [24]. For prevalence above 10%, we will stratify OR and RR by study type.

Appropriate data will be pooled using the program R and the “metafor” package [25]. The restricted maximum-likelihood estimator (REML) will be used for the calculation of grouped data and to explore and handle heterogeneity of datasets. We will apply the random effects model for taking into account the differing characteristics of participants and exposure across similar studies [26].

We will narratively synthesize evidence with estimations of RR or OR that is not suitable for a meta-analysis due to deficient comparability. Furthermore, a narrative synthesis is determined a priori for study results provided as hazard ratio, incidence rate ratio, cumulative incidence, and incidence rate.

Depending on the availability of data for meta-analysis we will form groups for separate calculations. We will separately synthesize outcome data for children and adults. The quantitative analysis for adults is further divided into groups for the occupational and the environmental setting. Additional divisions of the aforementioned groups will be undertaken according to the type of exposure. These types may be gases, fumes, dust, particular matter (PM) or man-made mineral matter. Further separation may be indicated by distinctive properties of substances for which available datasets could be found.

We will attempt to contact the authors of particular studies if summary data that are required for quantitative analyses are missing. If data for calculation of the standard error remain unavailable, we will qualitatively review the affected cohort and case-control studies with caution. Quality ratings will involve missing data and the discussion will address the impact of missing data on the findings.

The consistency of quantitative analyses will be visually explored in forest plots evaluating the relation of confidence intervals of effect measures from individual studies to each other. We will formally assess the degree of heterogeneity using the I^2^ and τ^2^ statistics. Heterogeneity is categorized as low, moderate and high at 25, 50 and 75% respectively [27]. Effects of covariates in meta-regression analyses will help to identify causes of heterogeneity.

#### Meta-regression and subgroup analyses

We will use meta-regression investigating the effect of several factors on heterogeneity of summary estimates that we determine a priori. These factors are the study type, the diagnostic standard and quality of exposure data. Apart from the pre-stated factors, we will conduct meta-regression including factors as covariates that will be identified in the course of systematic reviewing. Furthermore, particular attention will be paid to gender and co-exposures.

We may undertake subgroup analyses for datasets defined by distinctive properties of substances or mixtures for which available datasets will be found. If risk factors are provided in categories of different burdens of exposure, we will conduct subgroup analyses according to the burden.

#### Narrative summary

We will present narratively summarized evidence in the text and in tables. Results will be associated in groups according to the outcome and the kind of exposure, i.e., the particular causal substance, group of substances or similar mixtures.

#### Meta-biases

Risk of bias across studies will be assessed for each outcome using the GRADE (Grading of Recommendations, Assessment, Development and Evaluations) [28] framework and the corresponding instructions, which includes publication bias as a meta-bias. If we become aware of a more suitable method, we may switch to the alternative. The interpretation of funnel plots will help with the assessment of publication bias in quantitatively analyzed studies.

#### Confidence in cumulative evidence

The GRADE [28] framework will be used for the determination of the quality of evidence on outcome level. If we become aware of a more suitable method, we may switch to the alternative.

## Discussion

We began with the presented systematic review procedure after registration in PROSPERO, in September 2019. In particular, the registered electronic search and the subsequent title/abstract screening for relevance were conducted. The process arrived at the retrieval of the full texts that will be formally assessed for inclusion in the systematic review.

Reviewing the evidence for exposure-effects on new-onset disease, we will include incidence studies and incidence case-control studies. However, detecting them requires a comprehensive literature search. Therefore, we used comprehensive but reasonable terms according to our eligibility criteria for the electronic search. This resulted in a relatively high proportion of less likely relevant records. Since a preliminary search had detected many studies on therapy, we implemented specific MeSH terms for their removal. However, other irrelevant records that the final electronic search terms detected were much less contextually definable. Without sufficiently specific terms for their removal, we would have risked a considerable loss of sensitivity and thus relevant articles. Additionally, extended title/abstract screening caused by a broad search approach was not a critical burden for our systematic review group. Therefore, we made no attempt to further raise precision of the electronic search and, considering sensitivity and precision [29], gave more weight to its sensitivity.

Furthermore, the application of the electronic search strategy to three literature databases revealed a rather low overlap of about 20% of the detected records. This suggests fair coverage of available peer-reviewed articles with EMBASE, MEDLINE and Web of Science, which is in line with methodological recommendations [1, 29].

We will screen all reference lists of the retrieved full texts of primary studies, which increases coverage of the eligible literature [29]. Additonally, reference lists of systematic reviews and meta-analyses detected by the electronic search that are relevant to the objective will also undergo screening for articles.

We explained the eligibility of studies reporting incidence and/or measures of effect based on incidence in the protocol. The expected relative infrequency of such studies was confirmed by the first title/abstract screening. Therefore, we decided to screen all initial search results independently and in duplicate. This will reduce the risk of missing important information and the risk of reviewer bias.

Exposure assessment in particular studies can be complex. Besides methods for measuring substances in air, geospatial information technologies can be incorporated for different purposes, e.g., geographic information systems (GIS) for geostatistical interpolation techniques [30]. In fact, several studies investigating a relationship between air pollution and health outcomes, including a case-control study on asthma, used GIS [31–33]. Consequently, analysis of exposure assessment will be a challenge for the quality assessment in this review.

Our approach covers effects of industrial air pollution if studies reportedly attribute emissions to industry. It will be interesting how eligible studies identified and/or differentiated substances of industrial origin and their impact. Besides resource requirements of incidence studies, this might be a crucial aspect for the availability of eligible articles.

Moreover, we will include the current evidence on biological plausibility of health effects for interpreting the findings. Mechanistic studies will support the discussion of causal relationships indicated by the eligible observational studies [34–36].

By interpreting our findings we will take into consideration present knowledge including existing reviews and meta-analyses and point to the new aspects that our work adds. It can be assumed that our results can be used for appropriate future precaution and preventive measures.

## Supplementary Information

**Additional file 1.** PRISMA-P (Preferred Reporting Items for Systematic review and Meta-Analysis Protocols) 2015 checklist: recommended items to address in a systematic review protocol.

**Additional file 2.** Search strategy used in the systematic review.

## Data Availability

The supplementary material of the systematic review article will provide data extracted from primary studies.

## Abbreviations

ATS/ERS: American Thoracic Society/ European Respiratory Society
DALY: Disability-adjusted life years
DiMoPEx: Diagnosis, Monitoring and Prevention of exposure-related non-communicable diseases
EU-COST: European Union-European Cooperation in Science and Technology)
GRADE: Grading of Recommendations, Assessment, Development and Evaluations
FEV1/FVC: Forced expiratory volume in 1 s over forced vital capacity ratio
GINA: Global Initiative for Asthma Management and Prevention
GOLD: Global Initiative for Chronic Obstructive Lung Disease
LLN: Lower limit of normal
MeSH: Medical subject headings
NCD: Noncommunicable disease
NOS: Newcastle Ottawa Scale
NO_2_: Nitrogen dioxide
OR: Odds ratio
PM_2.5_: Particulate matter with a diameter of 2.5 μm or less
PRISMA: Preferred reporting items for systematic review and meta-analyses
PRISMA-P: Preferred reporting items for systematic review and meta-analysis protocols
PROSPERO: International database of prospectively registered systematic reviews
REML: Restricted maximum-likelihood estimator
RR: Risk ratio
SIGN: Scottish Intercollegiate Guidelines Network

## References

[1] Shamseer L, Moher D, Clarke M, Ghersi D, Liberati A, Petticrew M (2015). Preferred reporting items for systematic review and meta-analysis protocols (PRISMA-P) 2015: elaboration and explanation. BMJ.

[2] Liberati A, Altman DG, Tetzlaff J, Mulrow C, Gøtzsche PC, Ioannidis JPA (2009). The PRISMA statement for reporting systematic reviews and meta-analyses of studies that evaluate healthcare interventions: explanation and elaboration. BMJ.

[3] World Health Organisation (2018). Noncommunicable diseases.

[4] World Health Organisation (WHO) (2010). Global status report on noncommunicable diseases.

[5] World Health Organisation (WHO) (2016). Ambient air pollution: a global assessment of exposure and burden of disease.

[6] Wichmann F, Muller A, Busi L, Cianni N, Massolo L, Schlink U (2009). Increased asthma and respiratory symptoms in children exposed to petrochemical pollution. J Allergy Clin Immunol.

[7] Khreis H, Cirach M, Mueller N, de Hoogh K, Hoek G, Nieuwenhuijsen MJ (2019). Outdoor air pollution and the burden of childhood asthma across Europe. Eur Respir J.

[8] Khreis H (2017). Exposure to traffic-related air pollution and risk of development of childhood asthma: a systematic review and meta-analysis. Environ Int.

[9] Kogevinas M, Zock JP, Jarvis D, Kromhout H, Lillienberg L, Plana E (2007). Exposure to substances in the workplace and new-onset asthma: an international prospective population-based study (ECRHS-II). Lancet.

[10] Blanc PD, Annesi-Maesano I, Balmes JR, Cummings KJ, Fishwick D, Miedinger D (2019). The occupational burden of nonmalignant respiratory diseases. An official American Thoracic Society and European Respiratory Society statement. Am J Respir Crit Care Med.

[11] Baur X, Bakehe P, Vellguth H (2012). Bronchial asthma and COPD due to irritants in the workplace - an evidence-based approach. J Occup Med Toxicol.

[12] Richardson WS, Wilson MC, Nishikawa J, Hayward RS (1995). The well-built clinical question: a key to evidence-based decisions. ACP J Club.

[13] Rovira E, Cuadras A, Aguilar X, Esteban L, Borras-Santos A, Zock JP (2014). Asthma, respiratory symptoms and lung function in children living near a petrochemical site. Environ Res.

[14] Pearce N (2012). Classification of epidemiological study designs. Int J Epidemiol.

[15] Cambridge University Press (2020). Industry. Cambridge dictionary.

[16] Culver BH, Graham BL, Coates AL, Wanger J, Berry CE, Clarke PK (2017). Recommendations for a standardized pulmonary function report. An official American Thoracic Society technical statement. Am J Respir Crit Care Med.

[17] Chung KF, Wenzel SE, Brozek JL, Bush A, Castro M, Sterk PJ (2014). International ERS/ATS guidelines on definition, evaluation and treatment of severe asthma. Eur Respir J.

[18] Global Initiative for Asthma Management and Prevention (2019). 2019 GINA report, global strategy for asthma management and prevention.

[19] Global Initiative for Chronic Obstructive Lung Disease (2019). Global strategy for the diagnosis, management, and prevention of chronic obstructive pulmonary disease (2019 report).

[20] Qaseem A, Wilt TJ, Weinberger SE, Hanania NA, Criner G, van der Molen T (2011). Diagnosis and management of stable chronic obstructive pulmonary disease: a clinical practice guideline update from the American College of Physicians, American College of Chest Physicians, American Thoracic Society, and European Respiratory Society. Ann Intern Med.

[21] Scottish Intercollegiate Guidelines Network (SIGN), Healthcare Improvement Scotland (2020). Search filters.

[22] Ouzzani M, Hammady H, Fedorowicz Z, Elmagarmid A (2016). Rayyan-a web and mobile app for systematic reviews. Syst Rev.

[23] Wells G, Shea B, O'Connell D, Peterson J, Welch V, Losos M (2019). The Newcastle-Ottawa Scale (NOS) for assessing the quality of nonrandomised studies in meta-analysis.

[24] Shrier I, Steele R (2006). Understanding the relationship between risks and odds ratio. Clin J Sport Med.

[25] Viechtbauer W (2019). Package ‘metafor’: meta-analysis package for R version 2.1–0.

[26] Borenstein M, Hedges LV, Higgins JPT, Rothstein HR (2010). A basic introduction to fixed-effect and random-effects models for meta-analysis. Res Synth Methods.

[27] Higgins JP, Thompson SG, Deeks JJ, Altman DG (2003). Measuring inconsistency in meta-analyses. BMJ.

[28] Guyatt GH, Oxman AD, Vist GE, Kunz R, Falck-Ytter Y, Alonso-Coello P (2008). GRADE: an emerging consensus on rating quality of evidence and strength of recommendations. BMJ.

[29] Lefebvre C, Glanville J, Briscoe S, Littlewood A, Marshall C, Metzendorf MI, Higgins JP, Thomas J, Chandler J, Cumpston M, Li T, Page MJ (2019). Searching for and selecting studies. Cochrane handbook for systematic reviews of interventions.

[30] Zou B, Wilson JG, Zhan FB, Zeng Y (2009). Air pollution exposure assessment methods utilized in epidemiological studies. J Environ Monit.

[31] Gorai AK, Tchounwou PB, Tuluri F (2016). Association between ambient air pollution and asthma prevalence in different population groups residing in eastern Texas, USA. Int J Environ Res Public Health.

[32] Lindgren A, Stroh E, Bjork J, Jakobsson K (2013). Asthma incidence in children growing up close to traffic: a registry-based birth cohort. Environ Health.

[33] Houot J, Marquant F, Goujon S, Faure L, Honoré C, Roth MH (2015). Residential proximity to heavy-traffic roads, benzene exposure, and childhood leukemia-the GEOCAP study, 2002-2007. Am J Epidemiol.

[34] Thurston GD, Balmes JR, Garcia E, Gilliland FD, Rice MB, Schikowski T (2020). Outdoor air pollution and new-onset airway disease. An official American Thoracic Society workshop report. Ann Am Thorac Soc.

[35] Vedal S (1997). Ambient particles and health: lines that divide. J Air Waste Manag Assoc.

[36] Pope CA, Dockery DW (2006). Health effects of fine particulate air pollution: lines that connect. J Air Waste Manag Assoc.

