# Supplementary material for: Outdoor air pollution from industrial chemicals causing new onset of asthma or COPD: a systematic review protocol
Source: J Occup Med Toxicol. 2020 Dec 28;15:38. doi: 10.1186/s12995-020-00289-6 (PMC7768640; doi:10.1186/s12995-020-00289-6)
Supplement: Supplementary file 2 — Additional file 2. Search strategy used in the systematic review. [file 12995_2020_289_MOESM2_ESM.pdf]

Supplementary material
Search strategy used in the systematic review „Outdoor air pollution from industrial chemicals causing new onset of asthma or COPD"
Lux H, Baur X, Budnik LT, Heutelbeck A, Teixeira JP, Neumann E, et al.

Search strategy

We will search quantitative studies that include the eligible statistical parameters. No restriction of study types is included into the electronic search strategy. Validated filters, preferably functions provided by the databases, for studies on human subjects and English full texts will be applied. The search is limited to studies from 2000 to the current year and will be repeated 12 months after its first application for updating results.
The tripartite structure of the electronic search represents the three relevant concepts population/problem, exposure, and outcome of the PECO approach. We included search terms as text words derived from information on asthma-related substances provided by Leikauf et al. [1, 2].

The following electronic search strategy will be applied with the provider OVID to the literature database MEDLINE:

1. (exp asthma/ or exp Lung Diseases, Obstructive/ or Chronic obstructive pulmonary disease.ti,ab,kf. or COPD.ti,ab,kf. or Pulmonary obstruction.ti,ab,kf. or asthma.ti,ab,kf. or asthmatic$.ti,ab,kf.) not (exp Pharmacologic Actions/ or exp Pharmacy/ or exp Pharmacology/ or exp Therapeutics/)
2. limit 1 to (English language and humans and yr="2000 –Current")
3. (Air pollution/ or Environmental Exposure/ or exp Occupational Exposure/ or exp Waste products/ or exp Inorganic Chemicals/ or exp Organic Chemicals/ or Acid Rain/ or Petroleum Pollution/ or Biofuels/ or exp Fossil Fuels/ or exp Tars/ or Manure/ or (exp Particulate Matter/ not Tobacco Smoke Pollution/) or industrial.ti,ab,kf. or industry.ti,ab,kf. or industries.ti,ab,kf. or refinery.ti,ab,kf. or refineries.ti,ab,kf. or occupational.ti,ab,kf. or environmental.ti,ab,kf. or pollution.ti,ab,kf. or chemical?.ti,ab,kf. or ammonia.ti,ab,kf. or fertilizer?.ti,ab,kf. or fertiliser?.ti,ab,kf. or fume?.ti,ab,kf. or petroleum?.ti,ab,kf. or petrol?.ti,ab,kf. or petrochemical?.ti,ab,kf. or petrochemistr*.ti,ab,kf. or asthmagen*.ti,ab,kf. or irritant?.ti,ab,kf. or trichloroethane?.ti,ab,kf. or benzene?.ti,ab,kf. or ethylbenzene?.ti,ab,kf. or dichlorobenzene?.ti,ab,kf. or trichloroethylene?.ti,ab,kf. or tetrachloroethylene?.ti,ab,kf. or chloromethyl ether?.ti,ab,kf. or chloroform?.ti,ab,kf. or xylene?.ti,ab,kf. or styrene?.ti,ab,kf. or carbon tetrachloride?.ti,ab,kf. or toluene?.ti,ab,kf. or decane?.ti,ab,kf. or butanol?.ti,ab,kf. or perchloroethylene?.ti,ab,kf. or methyl isobutyl ketone?.ti,ab,kf. or dichloroethane?.ti,ab,kf. or ethoxyethyl acetate?.ti,ab,kf. or aldehyde*.ti,ab,kf. or formaldehyde?.ti,ab,kf. or propionaldehyde?.ti,ab,kf. or acetaldehyde?.ti,ab,kf. or acrolein?.ti,ab,kf. or isocyanate?.ti,ab,kf. or diisocyanate?.ti,ab,kf. or polyisocyanate?.ti,ab,kf. or anhydride?.ti,ab,kf. or hydrazine?.ti,ab,kf. or phenylenediamine?.ti,ab,kf. or dimethyl carbamyl chloride?.ti,ab,kf. or dimethyl sulfate?.ti,ab,kf. or phosgene?.ti,ab,kf. or phosphine?.ti,ab,kf. or chlorine?.ti,ab,kf. or hydrogen fluoride?.ti,ab,kf. or phthalate?.ti,ab,kf. or hydrochloric acid?.ti,ab,kf. or Hydrogen sulfate?.ti,ab,kf. or Hydrogen sulfide?.ti,ab,kf. or polycyclic aromatic?.ti,ab,kf. or dimethyl carbonyl chloride?.ti,ab,kf. or metal*.ti,ab,kf. or

cadmium?.ti,ab,kf. or chromium?.ti,ab,kf. or chromate?.ti,ab,kf. or cobalt?.ti,ab,kf. or nickel?.ti,ab,kf.) not (exp Pharmacologic Actions/ or exp Pharmacy/ or exp Pharmacology/ or exp Therapeutics/)

4. limit 3 to (English language and humans and yr="2000 –Current")
5. (Incidence/ or Odds ratio/ or exp Risk/ or relative risk.ti,ab,kf. or risk ratio.ti,ab,kf. or odds ratio.ti,ab,kf. or hazard ratio.ti,ab,kf. or incidence.ti,ab,kf. or FEV$1.ti,ab,kf. or FEV?1?.ti,ab,kf.) not (exp Pharmacologic Actions/ or exp Pharmacy/ or exp Pharmacology/ or exp Therapeutics/)
6. limit 5 to (English language and humans and yr="2000 –Current")
7. 2 and 4 and 6

The following electronic search strategy will be applied with the provider OVID to the literature database EMBASE:

1. (exp asthma/ or exp Lung Diseases, Obstructive/ or Chronic obstructive pulmonary disease.ti,ab,kw. or COPD.ti,ab,kw. or Pulmonary obstruction.ti,ab,kw. or asthma.ti,ab,kw. or asthmatic*.ti,ab,kw.) not (exp Pharmacologic Actions/ or exp Pharmacy/ or exp Pharmacology/ or exp Therapeutics/)
2. limit 1 to (English language and humans and yr="2000 –Current")
3. (Air pollution/ or Environmental Exposure/ or exp Occupational Exposure/ or Waste Products/ or exp Inorganic Chemicals/ or exp Organic Chemicals/ or Acid Rain/ or Petroleum Pollution/ or Biofuels/ or exp Fossil Fuels/ or exp Tars/ or Manure/ or (exp Particulate Matter/ not Tobacco Smoke Pollution/) or industrial.ti,ab,kw. or industry.ti,ab,kw. or industries.ti,ab,kw. or refinery.ti,ab,kw. or refineries.ti,ab,kw. or occupational.ti,ab,kw. or environmental.ti,ab,kw. or pollution.ti,ab,kw. or chemical?.ti,ab,kw. or ammonia.ti,ab,kw. or fertilizer?.ti,ab,kw. or fertiliser?.ti,ab,kw. or fume?.ti,ab,kw. or petroleum?.ti,ab,kw. or petrol?.ti,ab,kw. or petrochemical?.ti,ab,kw. or petrochemistr*.ti,ab,kw. or asthmagen*.ti,ab,kw. or irritant?.ti,ab,kw. or trichloroethane?.ti,ab,kw. or benzene?.ti,ab,kw. or ethylbenzene?.ti,ab,kw. or dichlorobenzene?.ti,ab,kw. or trichloroethylene?.ti,ab,kw. or tetrachloroethylene?.ti,ab,kw. or chloromethyl ether?.ti,ab,kw. or chloroform?.ti,ab,kw. or xylene?.ti,ab,kw. or styrene?.ti,ab,kw. or carbon tetrachloride?.ti,ab,kw. or toluene?.ti,ab,kw. or decane?.ti,ab,kw. or butanol?.ti,ab,kw. or perchloroethylene?.ti,ab,kw. or methyl isobutyl ketone?.ti,ab,kw. or dichloroethane?.ti,ab,kw. or ethoxyethyl acetate?.ti,ab,kw. or aldehyde*.ti,ab,kw. or formaldehyde?.ti,ab,kw. or propionaldehyde?.ti,ab,kw. or acetaldehyde?.ti,ab,kw. or acrolein?.ti,ab,kw. or isocyanate?.ti,ab,kw. or diisocyanate?.ti,ab,kw. or polyisocyanate?.ti,ab,kw. or anhydride?.ti,ab,kw. or hydrazine?.ti,ab,kw. or phenylenediamine?.ti,ab,kw. or dimethyl carbamyl chloride?.ti,ab,kw. or dimethyl sulfate?.ti,ab,kw. or phosgene?.ti,ab,kw. or phosphine?.ti,ab,kw. or chlorine?.ti,ab,kw. or hydrogen fluoride?.ti,ab,kw. or phthalate?.ti,ab,kw. or hydrochloric acid?.ti,ab,kw. or hydrogen

sulfate?.ti,ab,kw. or hydrogen sulfide?.ti,ab,kw. or polycyclic aromatic?.ti,ab,kw. or dimethyl carbonyl chloride?.ti,ab,kw. or metal*.ti,ab,kw. or cadmium?.ti,ab,kw. or chromium?.ti,ab,kw. or chromate?.ti,ab,kw. or cobalt?.ti,ab,kw. or nickel?.ti,ab,kw.) not (exp Pharmacologic Actions/ or exp Pharmacy/ or exp Pharmacology/ or exp Therapeutics/)

4. limit 3 to (English language and humans and yr=”2000 –Current”)
5. (Incidence/ or Odds ratio/ or Risk/ or relative risk.ti,ab,kw. or risk ratio.ti,ab,kw. or odds ratio.ti,ab,kw. or hazard ratio.ti,ab,kw. or incidence.ti,ab,kw.) not (exp Pharmacologic Actions/ or exp Pharmacy/ or exp Pharmacology/ or exp Therapeutics/)
6. limit 5 to (English language and humans and yr=”2000 –Current”)
7. 2 and 4 and 6

Electronic search strategy for Web of Science:

(TS=((asthma* or COPD or "Chronic obstructive pulmonary disease" or "pulmonary obstruction") and (pollut* or chemical$ or aerosol* or steam$ or vapo$r* or gas or gases or dust$ or fume$ or carbon or mineral$ or cement$ or waste$ or "acid rain" or "complex mixture$" or industr* or occupation$ or occupational or biofuel$ or "fossil fuel$" or tar$ or petrol* or petrochemical* or petrochemistr* or refinery or refineries or ammonia or fertilizer$ or fertiliser$ or manure or chlorine$ or trichloroethane$ or trichloroethylene or tetrachloroethylene$ or "chloromethyl ether$" or benzene$ or ethylbenzene$ or dichlorobenzene or chloroform or xylene$ or styrene$ or "carbon tetrachloride$" or toluene$ or decane$ or butanol$ or perchloroethylene$ or "methyl isobutyl ketone$" or dichloroethane$ or "ethoxyethyl acetate$" or aldehyde$ or formaldehyde$ or propionaldehyde$ or acetaldehyde$ or acrolein* or isocyanate$ or diisocyanate$ or polyisocyanate$ or anhydride$ or hydrazine$ or phenylenediamine$ or "dimethyl carbamyl chloride$" or "dimethyl sulfate$" or phosgene$ or phosphine$ or "hydrogen fluoride$" or phthalate$ or "hydrochloric acid$" or "hydrogen sulfate$" or "hydrogen sulfide$" or "polycyclic aromatic" or "dimethyl carbonyl chloride$" or metal$ or cadmium$ or chromium$ or chromate$ or cobalt$ or nickel$) and (incidence or "odds ratio" or "relative risk*" or "risk ratio" or “hazard ratio” or FEV?1? or FEV$))) AND LANGUAGE: (English) AND DOCUMENT TYPES: (Article)

We will apply the following filters and limitations to the searches in all databases if applicable:

1. English language
2. Humans
3. 2000 –Current

Additional to manual sorting, a validated filter of the SIGN initiative will help to identify systematic reviews and meta-analyses among initial search results in MEDLINE and EMBASE [3]:

Supplementary material
Search strategy used in the systematic review „Outdoor air pollution from industrial chemicals causing new onset of asthma or COPD"
Lux H, Baur X, Budnik LT, Heutelbeck A, Teixeira JP, Neumann E, et al.

MEDLINE

(Meta-Analysis as Topic/ or meta analy$.tw. or metaanaly$.tw. or Meta-Analysis/ or (systematic adj (review$1 or overview$1)).tw. or exp Review Literature as Topic/ or (cochrane or embase or (psychlit or psyclit) or (psychinfo or psycinfo) or (cinahl or cinhal) or science citation index or bids or cancerlit).ab. or ((reference list$ or bibliograph$ or hand-search$ or relevant journals or manual search$).ab. or ((selection criteria or data extraction).ab. and Review/))) not (Comment/ or Letter/ or Editorial/ or (animal/ not (animal/ and human/)))

EMBASE

((exp Meta Analysis/ or ((meta adj analy$) or metaanalys$).tw. or (systematic adj (review$1 or overview$1)).tw. or (cancerlit or cochrane or embase or (psychlit or psyclit) or (psychinfo or psycinfo) or (cinahl or cinhal) or science citation index or bids).ab. or (reference lists or bibliograph$ or hand-search$ or manual search$ or relevant journals).ab. or ((data extraction or selection criteria).ab. and review.pt.)) not ((letter or editorial).pt. or (animal/ not (animal/ and human/))))

References

1. Leikauf GD, Kline S, Albert RE, Baxter CS, Bernstein DI, Bernstein J, et al. Evaluation of a possible association of urban air toxics and asthma. Environ Health Perspect. 1995;103 Suppl 6:253-71.
2. Leikauf GD. Hazardous air pollutants and asthma. Environmental Health Perspectives. 2002;110 Suppl 4:505-26.
3. Scottish Intercollegiate Guidelines Network (SIGN), Healthcare Improvement Scotland. Search filters. 2020. https://www.sign.ac.uk/search-filters.html. Accessed 22 Jan 2020.
